# Genetic, epigenetic, and mechanistic studies of temporomandibular disorders and overlapping pain conditions

**DOI:** 10.1186/1744-8069-10-72

**Published:** 2014-12-15

**Authors:** Diane H Munzenmaier, Joan Wilentz, Allen W Cowley

**Affiliations:** Center for BioMolecular Modeling, Milwaukee School of Engineering, Milwaukee, WI 53202 USA; Science Editor, TMJ Association Ltd, Chevy Chase, MD 20815 USA; Department of Physiology, Medical College of Wisconsin, Milwaukee, WI 53226 USA

## Abstract

Leaders in the fields of Temporomandibular Disorders (TMD) and its accompanying overlapping pain conditions presented their latest findings at the Seventh Scientific Meeting of The TMJ Association, September 7–9, 2014, in Bethesda, MD. The meeting was co-sponsored by The TMJ Association and the National Institutes of Health. Topics of the scientific sessions included epidemiology and diagnostic criteria, basic mechanisms of chronic pain including the genetic and epigenetic basis of chronic pain, and the development of novel drugs for treatment of these conditions. Discussions were directed toward formulating a set of recommendations to advance research in this field.

## Epidemiology and diagnostics of TMD and overlapping pain conditions

Temporomandibular disorders are characterized by pain and dysfunction in one or both jaw joints and/or their associated bone, cartilage, and muscle tissues. Mild cases may resolve over time, but more severe and chronic cases occur with a number of other pain conditions at a higher frequency than would be expected by chance. These overlapping pain conditions include chronic headache, endometriosis, fibromyalgia, interstitial cystitis/painful bladder syndrome, irritable bowel syndrome, low back pain, myalgic encephalomyelitis/chronic fatigue syndrome, and vulvodynia. These disorders predominantly affect women in their child-bearing years, giving rise to moderate to severe pain and dysfunction in individuals over the course of a lifetime.

The presentations at the meeting emphasized that the diagnosis and treatment of TMD and overlapping pain conditions has progressed in very small increments over the past 50 years, citing at least four reasons: 1. TMD is currently associated with dentistry; however research indicates TMD to be a complex medical condition, best served through an interdisciplinary medical home. Currently, neither physicians nor dentists are effectively trained to address TMD. As a result there are no scientifically based standards of care or best practices. 2. The safety and effectiveness of any of the more than 50 treatments currently prescribed are unknown and in some cases can lead to iatrogenic complications that worsen the condition. 3. TMD and its overlapping pain conditions are stigmatized and patients, mostly female, are often not taken seriously. 4. The research, whether epidemiologic, basic, clinical or translational has been compartmentalized, focusing largely on the end organ affected. This has greatly hampered the sharing of diagnostic approaches and collaborative research which might lead to fresh insights into mechanisms and potential therapies.

Status reports on two major clinical studies were presented. The OPPERA (Orofacial Pain: Prospective Evaluation and Risk Assessment) study is a large, prospective study designed to identify risk factors for the development of TMD. Results of this study indicated that the observed rate of first-onset TMD in 2,737 participants with no history of TMD at enrollment was 3.5% per year. However, those who reported suffering from other pain conditions at the time of enrollment developed TMD at a significantly higher rate. The large-scale MAPP research network (Multidisciplinary Approach to the Study of Chronic Pelvic Pain) phenotyping study, is focused on characterization of patients with urologic chronic pain syndromes (e.g., interstitial cystitis/painful bladder syndrome) using multiple analytic measures including biomarkers, questionnaires, quantitative sensory testing (e.g., measuring pain threshold), and other neurobiological assessments. The goal of this multiple-level analysis is the identification of distinct patient subgroups, which might then lead to more targeted and improved treatment.

Emerging from the presentations and discussions was a critical need for resources to support research to elucidate the causes and mechanisms underlying TMD and overlapping pain conditions. Attendees urged that case definitions of these disorders be developed that conform to current scientific findings.

## Basic and clinical mechanistic studies in neuroscience and genomics

Multiple speakers discussed the diverse and interrelated mechanisms of TMD and chronic pain phenomena in general. Studies of pain processed by peripheral nociceptors (*nociceptive pain*) were presented showing that prolonged nociceptor activation leads to a peripheral sensitization consisting of increased action potential firing and neurotransmitter release in the dorsal horn. This peripheral sensitization in turn results in heightened excitability by dorsal horn neurons, known as central sensitization. The enhanced depolarization of these neurons causes recruitment of NMDA glutamate receptors leading to hyperalgesia and allodynia. In contrast, *neuropathic pain* produced by peripheral nerve lesions was shown to stimulate microglia in the dorsal horn with the release of modulators such as brain-derived neurotrophic factor, causing disinhibition of GABA/glycine inhibitory neurons. This in turn leads to central sensitization, similar to that observed in nociceptive pain.

Several investigators presented evidence that TRP ion channels, TRPV4 and TRPV1 in particular, are key mediators in pain transduction and peripheral sensitization in the trigeminal ganglion sensory neurons. It was proposed that TRP receptors should therefore be considered as potential drug targets for analgesics specifically targeting TMD and other related trigeminal pain disorders.

Also presented were novel observations showing that young patients with several types of chronic pain syndromes including fibromyalgia exhibit small-fiber polyneuropathies (SFPN) that can be objectively identified by skin biopsy. A novel, non-invasive confocal microscopy technique was presented for assessment of small fiber neuropathies such as reduced nerve length which is commonly found in fibromyalgia and other chronic pain conditions. Not only of diagnostic importance, these studies also suggested that early-onset SPFN has novel causes that can be treated by immunomodulatory therapy.

Discussions yielded a general consensus to broaden research in this area to advance understanding of the mechanisms leading to the chronicity of pain. Given the complexity of TMD and overlapping pain conditions, attendees recommended that there be better integration and coordination of research on all these conditions.

## Genetics, epigenetics, and functional genomics of TMD and overlapping pain conditions

Genome wide association studies (GWAS) conducted by OPPERA and MAPP investigators and reported at the meeting, have begun to reveal novel polymorphisms associated with chronic pain conditions. In contrast, work has only begun to assess genomic DNA modifications induced by environmental stressors that alter gene expression (i.e., epigenetics). One novel study presented used a neuropathic pain model (mouse spared nerve injury) to assess the effects of chronic pain on DNA methylation in the brain. After six months, methylation in prefrontal cortex and thalamus was reduced (in a reversible manner) suggesting a potential mechanism for changes in gene expression induced by chronic pain. Another study indicated that long-term opioid use led to increased DNA methylation associated with increased pain, thus offering an explanation for the development of opioid tolerance.

Attendees noted that the application of genomic scale approaches for the study of chronic pain requires the integration of large amounts of diverse types of phenotypic and genomic/sequence data. Intriguing work was presented on the construction of a global Bayesian causal network using genetic, epigenetic, and transcriptomic data to gain valuable insights into disease mechanisms and progression. It was clear that such quantitative predictive network models would be critical to advance this line of research.

Presenters discussed the use of exciting new technologies for gene editing, such as ZFN, TALE, and CRISPR/Cas9 methods to engineer both protein-based and RNA-guided transcriptional activators and repressors targeted to human genes that are already linked to pain pathways. An important research challenge in this field will be the development of novel methods for experimental and therapeutic delivery of engineered DNA-binding proteins.

In general, participants recommended that research be expanded to obtain molecular correlates of localized versus comorbid generalized chronic pain, anxiety, and depression. Genomic DNA and RNA deep sequencing is necessary to identify sequence variants and DNA methylation patterns that are associated with risk for overlapping pain conditions. Further, attendees saw a strong need to develop novel analytical tools to advance discovery in this field and for researchers to acquire and apply cutting-edge tools for targeted genome and epigenome editing.

## Development of therapeutics

Potential therapeutic pathways were presented by several investigators. One study targeted the inositol triphosphate receptor-1 by overexpression of an allosteric inhibitor, carbonic anhydrase-8 (Car8). This gene therapy resulted in blockade of the thermal hyperalgesia and mechanical allodynia exhibited in a Car8 null mutant mouse model. Another promising target was shown to be the metabotropic glutamate receptor 5. Preclinical studies on a negative allosteric modulator of this receptor, fenobam, have been encouraging, and it was proposed as a viable candidate for clinical trials as an analgesic.

Ranking among the highest priorities to emerge from the discussions at the meeting was a recommendation for the rapid development of novel, non-opioid, mechanistically based therapeutic agents for the treatment of the overlapping pain conditions under discussion. In this regard, participants recommended that efforts be broadened to identify novel molecular pathways and therapeutic targets that are amenable to manipulation and consequently can alter chronic pain modalities. Sensitive to research involving genome editing and other novel therapeutic approaches, attendees recommended that workshops be convened to address ethical issues and include patients, scientists, clinicians, legal experts, and ethicists.

## Conclusion

The Seventh Scientific Meeting of The TMJ Association focused on genetic, epigenetic, and mechanistic research using novel approaches. In addition to the participation of distinguished investigators from across the globe, the meeting was also attended by patients and advocates who were able to effectively and poignantly describe their long-time suffering and frustrations to the audience. Staff from a number of NIH Institutes and Centers attended the meeting and presented their view on how to address the challenges to translational research in this area and how their efforts might be coordinated. The broad focus of the meeting allowed for new interactions and fruitful discussions which will lead to new and innovative insights into the treatment of chronic pain conditions in the near future. To expedite progress, scientists and clinicians must work together with the private sector and federal agencies to build the infrastructure required for the recommended research and for its translation into clinical practice. NIH was urged to conduct a feasibility/cost analysis study to determine the funding and resources needed to support proposed epidemiological, mechanistic and genomic/epigenomic discovery research, together with new translational initiatives. This information could serve as a blueprint for the public, patient advocacy organizations, individual scientists and their professional organizations to alert Congress to the enormity of the chronic pain problem in the United States and the need to increase research funding on these chronic pain conditions. Research will result in improving the quality of health care and lives of the millions of people suffering.

